# The Role of ^123^I in the Management of Differentiated Thyroid Cancer: A Comprehensive Narrative Review

**DOI:** 10.3390/medsci14010068

**Published:** 2026-02-02

**Authors:** Pietro Bellini, Francesco Dondi, Michela Cossandi, Gianluca Viganò, Carlo Cappelli, Elisa Gatta, Davide Lombardi, Riccardo Morandi, Claudio Casella, Luigi Spiazzi, Carlo Rodella, Federica Saiani, Chiara Ingraito, Valentina Zilioli, Francesco Bertagna

**Affiliations:** 1 Nuclear Medicine, ASST Spedali Civili di Brescia, 25123 Brescia, Italy; 2 Nuclear Medicine, ASST Spedali Civili di Brescia and University of Brescia, 25123 Brescia, Italy; 3 Clinical Engineering, ASST Spedali Civili di Brescia, 25123 Brescia, Italy; 4 Department of Clinical and Experimental Sciences, SSD Endocrinologia, University of Brescia, ASST Spedali Civili, 25123 Brescia, Italy; 5 Otolaryngology Unit, ASST Spedali Civili di Brescia, 25123 Brescia, Italy; 6 Surgical Clinic, Università Degli Studi di Brescia and ASST Spedali Civili di Brescia, 25123 Brescia, Italy; 7 Health Physics Department, Spedali Civili, 25123 Brescia, Italy

**Keywords:** differentiated thyroid cancer (DTC), ^123^I scintigraphy, ^131^I scintigraphy, radioiodine (RAI), ^124^I PET/CT

## Abstract

Differentiated thyroid carcinoma (DTC) is the most common malignant endocrine tumor, with a generally favorable prognosis. Imaging, including iodine radioactive isotope scintigraphy (IRIS), is crucial for diagnosis and follow-up. While ^131^I has long been used for both therapeutic and diagnostic purposes, ^123^I is reserved for diagnostic imaging due to its shorter half-life and γ emissions. This review highlights the utility of ^123^I scintigraphy, especially in pre-treatment assessment and dosimetry for DTC. It is particularly valuable before radioiodine (RAI) ablation, providing accurate imaging in patients with iodine-refractory (IR) or biochemically incomplete response (BIR) DTC. When compared to post-therapeutic ^131^I scans, ^123^I scintigraphy appears to have a lower sensitivity for detecting metastatic lesions, particularly in lymph nodes and lungs. However, its diagnostic performance compared to low-dose diagnostic ^131^I is more variable, with some studies suggesting superiority due to the absence of stunning. Further research is needed to standardize its use and optimize its role in guiding DTC management.

## 1. Introduction

Differentiated thyroid carcinoma (DTC) is the most common malignant tumor of the endocrine system [1,2], and it presents a higher incidence in females than in males [3]. DTC is also generally characterized by a low incidence of distant metastases, which are the only features that greatly influence mortality and consequently a favorable prognosis [4,5,6,7]. Imaging examinations are fundamental in the staging and follow-up of the DTC, and they may include ultrasound (US), iodine radioactive isotope scintigraphy (IRIS) [8,9,10], and, in specific cases, ^18^F-fluorodesoxyglucose positron emission tomography-computed tomography (^18^F-FDG PET-CT) [11,12,13,14].

Since the 1940s, the IRIS represents the most specific and used diagnostic imaging examination in the management of DTC, with ^131^I and ^123^I as the most common isotopes. ^131^I has a half-life (T_1/2_) of about 8 days and emits low-penetrating β particles (maximum energy 606 kiloelectronvolts (keV), mean energy 190 keV). It is usually administered for DTC treatment [15,16]. However, due to its γ emissions, it can also be used to obtain whole-body scan (WBS) and single-photon emission tomography/computed tomography (SPECT/CT) images after both diagnostic and therapeutic doses [16,17,18]. ^123^I presents a prevalence of γ emission (primary emission of 159 keV) and a T_1/2_ of about 13 h, and it is used only for diagnostic imaging [17,18,19]. Despite being included in the most recent and relevant guidelines for the management of DTC [20], the exact role of ^123^I scintigraphy—and the clinical contexts in which it may be particularly useful—has yet to be clearly delineated.

A significant concern with using ^131^I for diagnostic scanning is the potential ‘stunning’ effect, where the diagnostic dose of ^131^I can impair the subsequent uptake of the therapeutic dose, potentially reducing treatment efficacy. Due to its shorter half-life and the absence of high-energy β emissions, ^123^I is theoretically free from this effect, making it an attractive agent for pre-therapeutic planning. However, its integration into clinical practice is heterogeneous, and its diagnostic performance relative to ^131^I, both in diagnostic and therapeutic settings, requires clarification.

The aim of this comprehensive narrative review is to summarize the current evidence regarding the role of ^123^I scintigraphy in dosimetry and pre-treatment RAI planning and to evaluate its diagnostic performance, particularly in comparison with ^131^I scintigraphy using diagnostic and therapeutic doses.

## 2. Radioiodine and Dosimetry

To quantify the red marrow absorbed dose after ^131^I administration, the 2008 European Association of Nuclear Medicine (EANM) procedure guidelines [21] recommend collecting serial blood samples at 2, 6, 24, 96, and 144 h following the administration of a low diagnostic activity (≤15 MBq), using blood activity as a surrogate for red marrow dosimetry and applying a safety threshold of 2 Gy. In addition, whole-body measurements are advised at 2, 6, and 24 h, with at least one further acquisition at approximately 96 h.

Hänscheid et al. [22] subsequently introduced a simplified method for estimating the blood absorbed dose per unit of administered ^131^I, relying on a single total-body retention measurement. This approach achieves its greatest accuracy when the measurement is acquired at 24–48 h and appears adequate for routine assessment of individual radiation exposure during radioiodine therapy, although it may carry a risk of underestimating the Maximum Tolerated Activity (MTA).

More recently, Atkins et al. [23] described a bi-exponential model corrected for body surface area (BSA), showing that the 48 h fractional whole-body retention can predict the measured MTA without requiring blood sampling, with an average deviation of only ±1.2%. The authors recommend administering no more than 70% of the calculated MTA, due to the risk of overestimation—particularly in patients prepared with rhTSH: this can occur due to faster RAI biological washout in patients prepared with rhTSH than in patients treated with hormone withdrawal; particularly, it be due to constipation and mild reduction of kidney function, the two main ways of RAI excretion through feces and urine, which are frequent during hypothyroidism.

Despite the increasing use of ^123^I scintigraphy, often combined with multiple SPECT/CT acquisitions, to determine the optimal therapeutic activity of ^131^I, the timing and methodology of ^123^I-based dosimetry remain non-standardized [24,25,26].

In preclinical work, Kim et al. [24] investigated the optimal timing for determining tissue-absorbed dose in DTC xenograft mouse models. They identified two key imaging time-points, at the estimated Tmax of 2.91 h and at 26 h following ^123^I administration.

Following the recommendations of Atkins et al., Durski et al. [25] tested a protocol involving two scintigraphic acquisitions at 24 and 48 h after ^123^I administration to estimate the ^131^I MTA in a patient with metastatic DTC; subsequently, 70% of the calculated MTA was administered.

Brown SR et al. [26] conducted a clinical trial investigating the potential role of selumetinib in promoting redifferentiation in DTC. The protocol incorporated ^123^I scintigraphy both for patient selection and for pre-treatment dosimetry in ^131^I therapy. Although the precise imaging schedule was not reported, the authors indicated that three to four acquisitions were performed, consisting of a standard scan followed by two or three additional time-point measurements.

Finally, the 2025 American Thyroid Association (ATA) guidelines for DTC management [20] also do not provide a standardized protocol for ^123^I-based dosimetry.

In summary, while ^131^I-based dosimetry is relatively well established, ^123^I dosimetry represents a promising and more patient-friendly alternative that eliminates the need for multiple blood samples. Emerging protocols—often based on two imaging time-points (e.g., 24 and 48 h)—appear feasible for estimating the MTA for ^131^I therapy, particularly in metastatic disease. However, the lack of a standardized ^123^I dosimetry procedure in the 2025 ATA guidelines highlights the need for larger, prospective studies to validate and harmonize these methods before they can be broadly implemented in clinical practice.

## 3. ^123^I WBS in Pre- RAI Treatment: Comparison with ^131^I Diagnostic Dose Scintigraphies

As reported in the recent 2025 ATA guidelines [20], the use of WBS prior to RAI therapy appeared to be a possible procedure to guide the treatment, preferably the use of WBS with ^123^I rather than WBS with ^131^I. Possible changes determined by WBS include the following:

(1) Detection of very large thyroid remnants, which require additional surgery prior to ablation;
(2) Absence of a remnant with negative thyroglobulin (Tg), particularly in patients at low risk of recurrence who do not require RAI therapy or who may require a low dose of RAI in other cases;
(3) Detection of RAI-avid metastases, offering the opportunity for high-dose treatment. Instead, the strongest arguments against this practice are provided by the 2008 EANM guidelines, which suggest not performing diagnostic WBS in the presence of a clear indication for RAI therapy, and the 2022 ETA guidelines, which state that diagnostic WBS should not be used routinely [27,28]. In addition, multiple studies analyzed its usefulness and concluded that, in several cases, ^123^I WBS findings modified the therapeutic approach [29,30,31]. In some cases, this involved making a conservative decision in the absence of ^123^I uptake; in others, it implies increasing the ^131^I dose administered in the presence of suspected metastases. ^123^I appeared to be favored over ^131^I due to its pure gamma emission, shorter half-life, and lower probability of stunning effect. In this context, it is interesting to evaluate the experience of using ^123^I WBS compared to ^131^I diagnostic WBS, considering the studies published in the literature.

Sarkar SD et al. [32] retrospectively reviewed 12 cases of patients who underwent thyroidectomy for DTC and compared the results of ^123^I whole-body scintigraphy (WBS), performed 24–96 h after administering 2–5 mCi (74–185 MBq) of ^123^I, with those of ^131^I WBS, performed at the same time points using a diagnostic dose of 111–185 MBq. They found that both ^123^I and ^131^I WBS correctly identified residual thyroid tissue in nine patients, but only ^131^I WBS detected metastatic lesions in five scans from four patients. No lesion was more clearly visualized with ^123^I than with ^131^I, leading the authors to conclude that ^123^I WBS is less sensitive than ^131^I WBS for imaging DTC metastases. The study’s limitations included the small sample size and the lack of single-photon emission computed tomography (SPECT/CT) imaging.

Mandel SJ et al. [33] prospectively evaluated 14 patients with DTC who underwent total thyroidectomy and were subsequently staged using ^123^I and ^131^I diagnostic scintigraphy. For the ^123^I WBS, images were acquired five hours after the administration of 48–56 MBq of ^123^I, and for the ^131^I scintigraphy, images were acquired 48 h after the administration of 111 MBq of ^131^I. They found that ^123^I scintigraphy detected three more foci of increased uptake than ^131^I WBS (35 versus 32 foci). The study’s limitations include the small sample size and the absence of SPECT/CT image acquisition.

Siddiqi A et al. [34] prospectively evaluated 12 patients with DTC previously treated with thyroidectomy and RAI and with negative ^131^I diagnostic scan and raised Tg levels. They performed a ^123^I scan on all the patients before RAI therapy, revealing positive findings in 10 out of 12 patients. A fundamental limitation was the selection bias, considering only patients with a negative ^131^I scan.

The comparison between ^123^I and diagnostic-dose ^131^I WBS yields conflicting results, as summarized in Table 1. The study by Mandel et al. [31], which reported superior performance for ^123^I, utilized an early acquisition time (5 h), potentially capitalizing on ^123^I’s favorable early pharmacokinetics. In contrast, Sarkar et al. [30] used later acquisitions (24–96 h), which may disadvantage ^123^I due to its physical short half-life. This highlights that the timing of image acquisition is a critical and often overlooked variable that significantly influences the perceived sensitivity of ^123^I scintigraphy. In addition, there are specific limitations that affect both RAI WBS with diagnostic dose. These include the potential for restricted RAI uptake in small thyroid remnants, the high cost of ^123^I, the possibility of misinterpretation of thyroid remnants as nodal metastases on planar images, and, finally, the limited sensitivity in the presence of unfavorable mutations (e.g., BRAF, TERT) [20].

## 4. ^123^I Diagnostic Dose vs. ^131^I Therapeutic Dose Scintigraphies

Several studies have attempted to compare the diagnostic performance of ^123^I WBS and ^131^I WBS after RAI therapy, reaching conflicting conclusions [35,36,37,38,39,40,41,42,43,44,45,46]. Most of the studies describe lower sensitivity of ^123^I WBS, with the possibility of misrecognizing some RAI-avid lesions that are detectable with ^131^I WBS [35,37,38,39,42,44]. However, other studies do not confirm these findings [40,41,43,45,46] and report comparable sensitivities, albeit often with lower values for ^123^I WBS than for ^131^I WBS. This would seem to confirm the latter’s slight superiority. Only one study described superiority of the ^123^I scan [36].

Considering single study, Alzahrani AS et al. [35] performed a retrospective study which compared 238 diagnostic WBS performed 24 h after the oral administration of 185–555 MBq of ^123^I with their corresponding ^131^I post-therapy WBS obtained 4–5 days after ^131^I therapy. They studied scans in three clinical situations: with the first ^131^I therapy, with the second ^131^I therapy, and in cases of elevated Tg levels and negative diagnostic scan. They found that 177 pairs were obtained with the first ^131^I therapy and showed concordance between pre-treatment and post-treatment scans in 166 pairs with a concordance rate (CR) of 93.8%: particularly, six post-treatment scans showed foci in thyroid bed and five in metastatic locations. Considering the second ^131^I therapy, 34 pairs were obtained and showed concordance in 28 pairs with a CR of 82.4%: particularly, in six cases, metastases were not detected with ^123^I WBS. Finally, of twenty-seven pairs of scans in patients with elevated Tg levels and negative pre-treatment WBS, fifteen post-treatment scans remained negative, six showed an uptake in the thyroid bed, three showed benign lung uptake, and three showed definite uptake (in thyroid bed, in thyroid bed and lung, and in lymph nodes) which was also weakly detectable retrospectively in the ^123^I WBS: CR in this setting was 55.6%.

Thomas DL et al. [36] retrospectively evaluated and compared pretherapy diagnostic ^123^I scans with 7-day post-therapy ^131^I scans in detecting remnant thyroid disease as well as locoregional metastases in 53 patients. They found that ^123^I scans performed 24 h after an oral administration of 1–1.6 mCi (37–59.2 MBq) were more sensitive and provided a better lesion-to-background ratio than ^131^I scans performed seven days after ^131^I oral administration. The study was probably affected by the late acquisition of the ^131^I WBS, and the authors acknowledge this in their conclusion.

Cohen JB et al. [37] retrospectively compared 30 pre-therapy diagnostic ^123^I scans performed 24 h after the administration of 74 MBq of ^123^I with 30 post-therapy ^131^I scans performed 2–10 (mean 5.8) days after the administration of RAI in 29 patients. They found that WBS showed concordance in nineteen cases (63.3%), while in four cases (13.3%), ^123^I WBS detected more lesions in the neck than ^131^I WBS. However, in seven cases (23.3%), ^131^I WBS were more sensitive than ^123^I WBS, particularly in cases involving distant metastases.

Bravo PE et al. [38] retrospectively evaluated 342 individuals diagnosed with DTC, underwent RAI therapy, and performed a ^123^I WBS one day before RAI administration and ^131^I WBS one week after RAI therapy. SPECT/CT acquisitions were also performed, and the administered ^123^I activities were standardized: 1.5 mCi for patients treated with thyroid hormone withdrawal and 2.0 mCi for patients prepared with recombinant human TSH (rhTSH). The patients were divided into three groups: 311 patients RAI-naive without known distant metastatic disease, 23 patients with history of prior RAI and persistent disease, and 8 patients with known distant metastases. Their analysis showed that 22 patients (7%) in the first group had discordant scan findings but in only four cases did these discrepancies reflect actual disease. In the second and third groups, discordant scans were present in seven (30%) and five (62.5%) patients, respectively, with misinterpretation of metastatic disease at the ^123^I WBS scan. Bravo PE et al. concluded that the sensitivity of ^123^I WBS depends on the clinical setting.

Iwano S et al. [39] published a study evaluating 69 patients with DTC treated with RAI. They compared WBS images taken 24 h after the administration of 37 MBq of ^123^I with images obtained 34 days after administration of 2.22–7.4 GBq (median 5.55 GBq) of ^131^I. They identified 108 sites of RAI uptake in the ^131^I WBSs, but only 77 of these were also detected with the ^123^I, giving a CR of 71%. CR was higher for uptake in the thyroid bed (89%) and bone metastases (86%) but lower for lymph nodes (61%) and lung metastases (39%). The authors concluded that WBSs performed 24 h after the administration of 37 MBq of ^123^I were not always predictive of the ^131^I WBS results.

Urhan M et al. [40] evaluated 292 ^123^I WBS (dose administered: 50–111 MBq) with their corresponding post-treatment ^131^I images. All patients treated with RAI were in a hypothyroid state. They found that in 228 out of 263 patients with a positive diagnostic scan, ^123^I and ^131^I WBS findings were concordant (CR 87%). However, 44 additional foci of abnormal uptake in 22 discordant cases were found in ^131^I WBS but with no impact on therapeutic management of the patients. In the other 13 patients, there was at least one new site on post-treatment images that had been missed on pre-treatment ^123^I images. Finally, 29 patients with a negative diagnostic WBS were treated with ^131^I due to high Tg levels (range 11.3–480 ng/mL) with RAI uptake sites not detected with ^123^I WBS seen in eight post-treatment ^131^I scans (CR 72%). The authors concluded that in DTC management pre-treatment, ^123^I WBS is comparable to high-dose ^131^I post-treatment imaging, but data apparently do not support this conclusion: in fact, 43 of 292 WBS (14.7%) had at least an RAI-avid lesion not detected with ^123^I WBS.

Gulzar Z et al. [41] prospectively evaluated 27 patients who underwent near total thyroidectomy and performed subsequent RAI therapy. They compared WBS obtained 4 and 24 h after the administration of 185 MBq ^123^I, with WBS performed 5–7 days after administration of therapeutic dose of ^131^I. They found CR of 92.6% and 83.2%, respectively, for WBS performed after 4 and 24 h following ^123^I administration compared with ^131^I WBS. Particularly, 7.8% of patients had at least one RAI-avid lesion that was not detected with the ^123^I WBS.

De Geus-Oei LF et al. [42] studied 55 patients who had been treated with ^131^I (1.85–5.55 GBq) and had previously undergone diagnostic ^123^I WBS (111–370 MBq). They compared WBS obtained 24 h after ^123^I administration with WBS obtained 3–15 days (median 8.6 days) after ^131^I administration in the 36 patients for whom these data were available. They found that more lesions were visible on the post-therapeutic ^131^I WBS than on the corresponding diagnostic ^123^I scan in thirteen patients; in two patients, ^123^I WBS appeared more sensitive; and in twenty-one, the findings were equal (CR 58.3%).

Ali N et al. [43] retrospectively evaluated 58 patients who underwent at least two ^131^I therapy and made a comparison between a 20 min scintigraphy obtained 2 and 24 h after the administration of ^123^I 185–270 MBq and a 10–12 min scintigraphy obtained 4–7 days after the administration of 5.55 GBq of ^131^I. The study, which initially included 135 patients, showed that despite a high complete response rate (94.8%), more lesions were visible on the post-therapeutic ^131^I WBS than on the corresponding diagnostic ^123^I scan. Notably, in three patients, the ^123^I WBS was negative while the ^131^I scan was positive. In one patient, ^123^I scintigraphy detected an uptake that was not seen on the ^131^I scan; however, a subsequent scan performed after the third ^131^I therapy eventually revealed it.

Siddiqi A et al. [34], as previously reported, prospectively evaluated 12 patients with DTC previously treated with thyroidectomy and RAI and with negative ^131^I diagnostic scan and raised Tg levels (>2.5 pg/L): in these patients, a diagnostic ^123^I WBS was performed 2 h and 24 h after administration of ^123^I tracer dose 185 MBq and 4–7 days after ^131^I therapy dose 5.55 GBq. They performed a total of 18 ^123^I scans and found 16 positive ^123^I WBS with corresponding 14 positive ^131^I WBS: in the two cases of discordance, one was a false negative uptake; in the second one, the Tg levels decreased after therapy; and no imaging or histopathological confirmation of the bone uptake at ^123^I scan was performed. In two cases, ^131^I WBS detected thyroid bed uptakes not revealed by ^123^I. The primary limitation of the study—aside from the small sample size and the absence of SPECT/CT images—was the patient selection criterion, which included only patients with a negative ^131^I diagnostic WBS.

Schoelwer MJ et al. [44] retrospectively evaluated 33 pediatric patients who performed total thyroidectomy, diagnostic scintigraphy, and RAI therapy for DTC. Thirty-seven pairs of scans with different iodine isotopes were performed. For diagnostic scanning, five received 74 MBq of ^131^I, twenty-one received 74 MBq of ^123^I, and eleven received 111 mCi of ^123^I; images were acquired 24 h after RAI administration. Therapeutic dose of ^131^I was variable (27–190 mCi) and images acquired after 7 days. Of the 31 ^131^I scans considered, 24/31 were concordant with ^131^I therapeutic scans (CR 77%), particularly in two cases with missing mediastinal uptake and four cases with missing lung uptake (false negative ^123^I rate: 19.4%). One case was represented by a false positive ^131^I finding.

Yaakob W et al. [45] retrospectively evaluated 13 patients with DTC who underwent thyroidectomy and were given ^123^I whole-body scans 24 h after receiving 0.8–1.0 mCi of ^123^I. All patients then received a therapeutic dose of ^131^I (29.9–250 mCi), after which a WBS was performed 7–10 days later. Excluding one patient with a false positive ^123^I WBS scan, the authors found excellent correlation between scans in 11 out of 12 patients, particularly in the case where ^123^I underestimated the lung burden (CR 91.7%).

Berbano R et al. [46] conducted a study involving 16 patients with DTC treated with RAI. They compared whole-body scans performed 24 h after administration of 10 mCi (370 MBq) of ^123^I with images acquired 5–7 days after ^131^I administration (dose range: 75–200 mCi). They observed concordant findings in 15 of the 16 scans (CR 93.8%), with a single discordant case in which the ^123^I WBS failed to detect a lung lesion.

Most of the studies were affected by the limitation of the absence of SPECT/CT images, resulting in low diagnostic performance of the scintigraphic images. Furthermore, some studies [34,38,41,44,45,46] had a small sample size (<30 patients). Another possible source of bias is the heterogeneity in the ^123^I scintigraphy protocol, including the administered dose and timing of image acquisition. A similar problem is also present for ^131^I scintigraphy after a therapeutic dose. Finally, excluding the paper by Gulzar Z et al. [41], most studies had a retrospective design.

Table 2 summarizes the findings of the studies reported in the comparison of ^123^I diagnostic scintigraphy and scintigraphy performed after a therapeutic dose of ^131^I.

**Figure 1:**
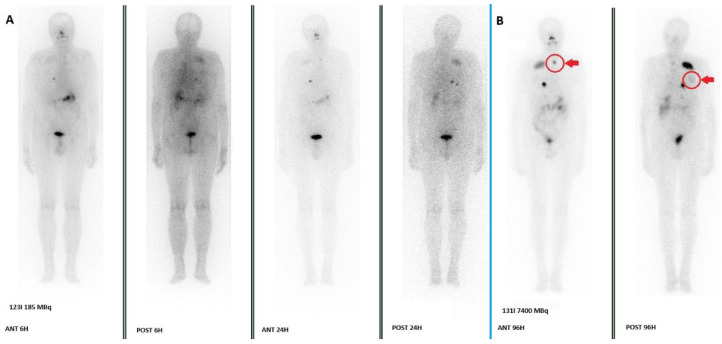
A comparison of a WBS performed 6 and 24 h after the injection of 185 MBq of ^123^I (**A**) and a WBS performed 96 h after RAI therapy (7400 MBq) (**B**) was conducted in a patient with known bone and pulmonary metastases who was treated in our department in 2024. The images show the detection of a new nodal localization in the neck (arrow and circle in ANT 96H) and weak uptake in known rib metastases (arrow and circle in POST 96H) in the ^131^I WBS, whereas only faint and unclear uptakes were present in the ^123^I WBS.

The bulk of evidence from Table 2 indicates that post-therapeutic ^131^I WBS detects a higher number of RAI-avid lesions than pre-therapeutic ^123^I WBS. The concordance rates are generally high in treatment-naïve patients with low tumor burden but drop significantly in the setting of persistent or metastatic disease. The most common sites for ^123^I false negatives are lymph nodes and pulmonary metastases, likely due to lower lesion uptake and poorer count statistics compared to the high activity used in therapy. Therefore, a negative ^123^I scan in a high-risk patient, particularly one with elevated Tg levels, should be interpreted with caution, as it does not definitively exclude the presence of RAI-avid disease. Figure 1 shows an example of WBSs confrontation in a patient who was treated for metastatic DTC.

## 5. ^123^I Scan and Thyroglobulin Level

Tg is a protein produced only by follicular thyroid cells. After thyroidectomy and, in particular, RAI ablation, it represents a fundamental tumor marker for the management and follow-up of DTC [47]. Its level and/or rising trend are well known to be associated with disease recurrence, as reported in the ATA 2025 guidelines. They defined dynamic risk stratification classes according to the response to therapy and the Tg levels. This includes, other than an excellent response (ER) and a structural incomplete response (SIR), an indeterminate response (IR) in the presence of a basal Tg (bTg) range of 0.2–1.0 ng/mL and a biochemically incomplete response (BIR) in the presence of a bTg range of 1.0–10.0 ng/mL, which is associated with an increased risk of structural recurrence of the disease. In this context, some studies have analyzed the usefulness of ^123^I scintigraphy in relation to Tg levels [48,49,50,51].

Campennì et al. [48] conducted a retrospective evaluation of 124 patients with DTC who underwent thyroidectomy and RAI therapy during follow-up. The study focused on the utility of the ^123^I scan. They found that the diagnostic performance of ^123^I WBS SPECT/CT significantly increased in patients with basal Tg values of at least 0.39 ng/mL.

Sol B et al. [49] retrospectively evaluated 40 patients with DTC who had undergone total thyroidectomy, focusing on 24 patients with undetectable basal Tg level determined with a highly sensitive Tg assay (below 0.1 ng/mL) six months after thyroidectomy. They found that none of these patients had stimulated Tg level above 1 ng/mL or a remnant on the ^123^I WBS after one year of follow-up. They concluded that ^123^I scintigraphy is unnecessary in this patient group.

Again, Campennì et al. [50] retrospectively evaluated 241 low- to intermediate-risk patients with histologically confirmed DTC and negative Tg antibodies (AbTg) all treated with total thyroidectomy and RAI therapy. During the follow-up 8–12 months after RAI, a ^123^I WBS was performed in 51 patients, particularly in 16 patients without excellent response. They found that seven out of sixteen (43.7%) DTC patients with IR (mean stimulated Tg = 4.5 ng/mL) and/or BIR (mean stimulated Tg = 13.5 ng/mL) ^123^I WBS and SPECT/CT were able to identify five local and two locoregional lymph-node metastases.

Villani MF et al. [51] evaluated 55 pediatric patients with DTC, and, particularly, they considered 41 scans performed after ^123^I administration with rhTSH and before RAI therapy. They found that thyroglobulin alone apparently was not a good predictor for staging modification (AUC = 0.6855 in ROC analysis), while ^123^I WBS modified staging in 12/41 (29%): in 3/12 (25%) for the presence of lung metastases and in 9/12 (75%) for lymph node involvement. In all these patients, the therapeutic management were modified.

Table 3 summarizes the findings of the studies reported.

## 6. Discussion

^123^I scintigraphy remains a key tool in the management of DTC, in particular in the diagnostic setting during the follow-up, especially in patients with IR and/or BIR as reported by Campennì A et al. [48,50], although its sensitivity is limited [35,38]. Its possible utility in performing it after total thyroidectomy and prior to RAI therapy has also been reported [29,30,31,51]. Execution of ^123^I diagnostic images allows the assessment of metastatic DTC localization or can underline the presence of unexpected alterations of radioiodine distribution in several cases [28]. These findings can help to define the appropriate RAI dose, if indicated, or can modify the therapeutic approach to these subjects: this applies to both initial therapy after total thyroidectomy and follow-up therapy.

In contrast with its utility in cases of dosable bTg, the absence of incremental diagnostic value in the presence of negative bTg was also described in one study. Particularly, the study of Sol B et al. [49] reported that the presence of bTg < 0.1 ng/mL during the early follow-up after initial therapy apparently made unuseful the ^123^I scintigraphy. These data appear to agree with the literature and particularly with the high negative predictive value of the bTg data [52,53]. Therefore, there is no benefit in routinely performing 123I WBS during the follow-up of patients with ER.

It is important to highlight that studies comparing ^123^I and ^131^I scintigraphy reported in the literature describe an apparent superiority of the ^123^I diagnostic scan over the ^131^I scan [33,34], while ^131^I scans performed after RAI therapy appear to be more sensitive than ^123^I diagnostic WBS scans [35,37,38,39,42,44]. Studies reporting comparable diagnostic values for ^131^I WBS after RAI therapy and ^123^I diagnostic WBS mostly describe a slight superiority of the former. In particular, few patients in these studies had a DTC lesion missing on the ^123^I WBS that was subsequently detected with the ^131^I. Urhan et al. [40] reported this to be 14.7%, Gulzar et al. [41] 7.8%, Ali et al. [43] 5.2%, Yaakob et al. [45] 8.3%, and Berbano et al. [46] 6.2%. Given that the presence of metastases is one of the few factors that can significantly affect outcomes in patients with DTC [54], the possibility of missing them in 5.2–14.7% of cases warrants caution when using ^123^I diagnostic scintigraphy during follow-up. In summary, ^123^I WBS scintigraphy appears to provide useful information when positive results are reported. However, a negative result could not exclude the presence of a RAI-avid lesion in a few cases, despite its good sensitivity. In conclusion, the ^131^I WBS performed after the administration of therapeutic activity remains the gold standard for evaluating RAI avidity in the localization of DTC disease.

It is important to emphasize that no clear standardization protocol on ^123^I dose or acquisition time were described for patient dosimetry, even though diagnostic practice studies reported the administration of different ^123^I doses. Almost all these studies agreed on acquiring images 24 h after administration, whereas the utility of an earlier acquisition is still being debated. In the same context, no studies focused on comparing preparation with hormone withdrawal in relation to rhTSH were found in the literature. This lack of standardized protocols requires an expert consensus to establish a well-known and widely utilized practice, especially considering the large number of studies reported in the literature. Standardization of ^123^I WBS dosimetry and diagnostic protocols, including the dose, timing, and patient preparation, could provide a basis for further studies evaluating its usefulness and enabling better comparison.

**Figure 2:**
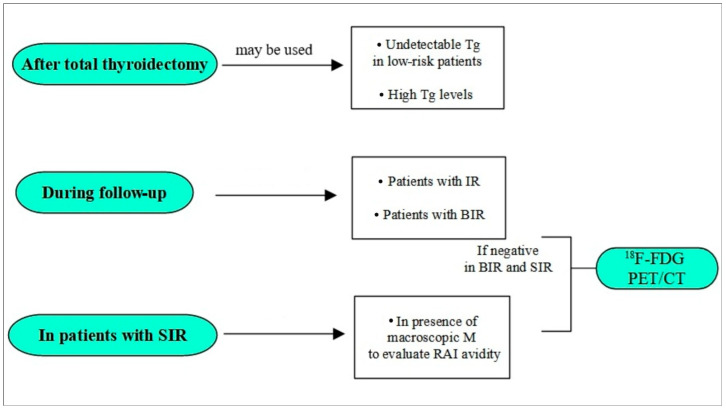
A flow chart about possible roles of ^123^I scintigraphy inside the DTC management.

Furthermore, it is fundamental to highlight that the choice of imaging in DTC is not merely between ^123^I and ^131^I, and performing an RAI WBS does not necessarily conclude the diagnostic process. In this setting, ^18^F-FDG PET/CT has a well-established role in RAI refractory DTC where lesions lose the ability to concentrate iodine [55,56]. A key clinical challenge is identifying which patients could benefit from ^18^F-FDG PET/CT and where ^123^I can serve as a triage tool. Considering patients with BIR, a positive scan confirms the presence of RAI-avid lesions and guides therapy, albeit with some limitations, while a negative scan should prompt investigation for iodine-refractory disease with ^18^F-FDG PET/CT. Similarly, in patients with known SIR, the possibility to evaluate both the RAI and FDG uptake in every single metastases could guide the therapeutic choice. Possible roles of the ^123^I scintigraphy in the management of DTC are highlighted in Figure 2.

Finally, beyond conventional planar or SPECT-based approaches, it is important to acknowledge the additional contribution that PET imaging can offer in the evaluation of iodine-avid disease. In this context, ^124^I PET/CT provides superior sensitivity and true quantitative capabilities, enabling improved detection of small-volume lesions—particularly cervical lymph node involvement and micronodular pulmonary metastases—and offering a more accurate basis for patient-specific dosimetry. Several studies [57,58,59] have demonstrated that ^124^I PET outperforms ^123^I scintigraphy in lesion conspicuity and may better predict post-therapeutic ^131^I uptake, supporting its potential role in treatment planning. Nevertheless, its routine clinical use remains limited by restricted availability and higher costs.

We can actually suggest the use of ^123^I scintigraphy for pre-ablation assessment in cases of unclear RAI indication, IR, BIR, and SIR, bearing in mind its limited sensitivity. This can be followed by an ^18^F-FDG PET/CT scan if the WBS is negative. Finally, ^123^I should be used for dosimetry when required.

The review has several limitations. Firstly, most of the studies appeared quite old and lacked SPECT/CT image acquisition, which is actually the gold standard for managing DTC as recommended by several international guidelines, including the recent SNMMI/EANM 2022 and ATA 2025 guidelines [20,60]. This could lead to an underestimation of the sensitivity of the two tracers considered. Secondly, the absence of standardization in administration and acquisition protocols resulted in heterogeneous use of dosage and acquisition time in ^123^I diagnostic scintigraphy. Similarly, significant heterogeneity in the ^131^I therapeutic dose administered to patients, as well as in the timing and acquisition protocol of the scintigraphic images, was reported. This heterogeneity makes it difficult to compare individual studies and surely affects the sensitivity of scintigraphic studies. Thirdly, most of the studies considered were designed with retrospective analyses. Finally, another important possible limitation is represented by the small sample size of some studies considered.

Further studies with a large sample size and a prospective design should be desirable to define the real discrepancy between ^123^I and ^131^I scintigraphy after RAI therapy. Other variable such as ^18^F-FDG PET/CT, Tg levels, the staging, the risk factor, etc., should be also considered in the analysis. This approach could confirm whether the latter is superior and identify patients in whom the possibility of a false negative result with ^123^I is present.

## 7. Conclusions

In conclusion, ^123^I scintigraphy represents a valuable diagnostic tool in the management of DTC in specific settings, particularly in cases of raised Tg levels (IR and/or BIR), patients with SIR, and for dosimetry in metastatic disease. Instead, its role in pre-ablation assessment is still debated. Moreover, it is fundamental to highlight its possible lower sensitivity compared to post-therapeutic ^131^I scans. The development of standardized protocols for administered activity and acquisition timing is needed. Future prospective, multi-center studies with large sample sizes, standardized ^123^I protocols, and mandatory SPECT/CT are essential to definitively establish its sensitivity and integrate it into a cost-effective, patient-tailored diagnostic algorithm.

## Figures and Tables

**Table 1:** Summary of articles comparing ^123^I with diagnostic-dose ^131^I WBS.

Reference	Authors	Year	Nation	N of Patients	Conclusion	Concordance Rate (CR) (%)	Possible Bias	Design
[32]	Sarkar SD et al.	2002	USA	12	^131^I more sensitive than ^123^I	61.5%	Small sample size, no SPECT/CT acquisition	Retrospective
[33]	Mandel SJ et al.	2001	USA	14	^123^I more sensitive than ^131^I	91.4% *	Small sample size, no SPECT/CT acquisition	Prospective
[34]	Siddiqi A et al.	2001	UK	12	^123^I more sensitive than ^131^I	nd **	Selection bias: study reported just a representation of the not unusual possibility of false negative of ^131^I diagnostic scan	Prospective

* number of foci. ** they selected only patients with negative ^131^I scan.

**Table 2:** Summary of articles that comparing ^123^I scintigraphy with therapeutic-dose ^131^I WBS.

Reference	Authors	Year	Nation	N of Patients	Conclusion	CR (%)	Possible Bias	Design
[34]	Siddiqi A et al.	2001	UK	12 *	^123^I comparable with ^131^I	nd	Selection bias. Absence of SPECT/CT. Small sample size.	Prospective
[35]	Alzahrani AS et al.	2001	Saudi Arabia	238	^131^I more sensitive than ^123^I	93.8 in first RAI therapy; 82.4 in second RAI therapy; 55.6 in patients with high Tg and negative pre-treatment WBS	Different doses of ^123^I and ^131^I administered. Absence of SPECT/CT	Retrospective
[36]	Thomas DL et al.	2009	USA	53	^123^I more sensitive than ^131^I	26.4	Late acquisition of ^131^I WBS. Different doses of ^131^I administered. Absence of SPECT/CT	Retrospective
[37]	Cohen JB et al.	2004	USA	29	^131^I more sensitive than ^123^I	63.3%	Different doses of ^131^I administered. Absence of SPECT/CT. Small sample size.	Retrospective
[38]	Bravo PE	2013	USA	342	^131^I more sensitive than ^123^I	93% in the first RAI therapy; 70% in patients previously treated and with persistent disease; 37.5% in patients with known M1	/	Retrospective
[39]	Iwano S et al.	2009	Japan	69	^131^I more sensitive than ^123^I	71%	Different doses of ^131^I administered. Low dose of ^123^I administered. Absence of SPECT/CT	Retrospective
[40]	Urhan M et al.	2007	USA	292	^123^I comparable with ^131^I **, but data suggest ^131^I more sensitive than ^123^I	85.3%	Different doses of ^123^I and ^131^I administered. Absence of SPECT/CT	Retrospective
[41]	Gulzar Z et al.	2001	USA	27	^123^I comparable with ^131^I **	92.6% in images after 24 h; 85.2% in images after 4 h	Different doses of ^131^I administered. Absence of SPECT/CT. Small sample size.	Prospective
[42]	De Geus-Oei LF et al.	2002	The Netherlands	55 ***	^131^I more sensitive than ^123^I	58.3%	Different doses of ^131^I administered. Absence of SPECT/CT. Small sample size.	Retrospective
[43]	Ali N et al.	2006	UK	58 ****	^123^I comparable with ^131^I **	94.8%	Retrospective nature of the study	Retrospective
[44]	Schoelwer MJ et al.	2015	USA	33 *****	^131^I more sensitive than ^123^I	77%	Different doses of ^131^I administered. Absence of SPECT/CT. Small sample size	Retrospective
[45]	Yaakob W et al.	1999	USA	13	^123^I comparable with ^131^I **	91.7%	Different doses of ^131^I administered. Absence of SPECT/CT. Small sample size.	Retrospective
[46]	Berbano R et al.	1998	USA	16	^123^I comparable with ^131^I **	93.8%	Different doses of ^131^I administered. Absence of SPECT/CT. Small sample size.	Retrospective

* 12 patients but 18 ^123^I and ^131^I WBS performed. ** Authors’ conclusion, but data may suggest ^131^I little better than ^123^I. *** Comparison between ^123^I and ^131^I WBS were available for 36 patients. **** Study enrolled 135 patients, but only 58 were retreated with ^131^I. ***** 33 patients enrolled, 37 WBS performed, but 5 excluded because of performed with ^131^I diagnostic dose and one because of false positive ^123^I result.

**Table 3:** Summary of articles evaluating the relationship between ^123^I scintigraphy and Tg levels.

Reference	Authors	Year	Nation	N of Patients	Design	Possible Bias	Conclusion
[48]	Campennì et al.	2023	Italy	124	Retrospective	Retrospective nature of the study	^123^I scintigraphy diagnostic value appeared higher in patients with bTg > 0.39 ng/mL
[49]	Sol B et al.	2021	Belgium	24 *	Retrospective	Small sample size, retrospective nature of the study	^123^I scintigraphy is not useful in patients with undetectable bTg (<0.1 ng/mL) 6 months after thyroidectomy
[50]	Campennì et al.	2021	Italy	16 **	Retrospective	Retrospective nature of the study	^123^I scintigraphy use appears very useful during follow-up of patients with DTC and BIR or IR
[51]	Villani MF et al.	2018	Italy	41 ***	Retrospective	Retrospective nature of the study	Tg alone give no strength information in staging modification prior to RAI therapy. ^123^I appears useful in the definition of therapeutic management of patients with DTC prior to RAI.

* 24 Patients with ER were selected from a sample of 40 patients. ** 16 Patients without ER were selected from a sample of 51 patients. *** 40 Patients who performed a WBS with 123I before RAI therapy were selected on a sample of 55 patients.

## Data Availability

No new data were created or analyzed in this study.
